# Exploring current and potential roles of Australian community pharmacists in gout management: a qualitative study

**DOI:** 10.1186/s12875-018-0744-3

**Published:** 2018-05-09

**Authors:** Allyce B. Counsell, Amy D. Nguyen, Melissa T. Baysari, Diluk R. W. Kannangara, Andrew J. McLachlan, Richard O. Day

**Affiliations:** 1 Department of Clinical Pharmacology & Toxicology, St Vincent’s Hospital, Sydney, NSW Australia; 2 School of Medical Sciences, University of New South Wales, Sydney, NSW Australia; 3 St Vincent’s Clinical School, St Vincent’s Hospital, University of New South Wales, Sydney, NSW Australia; 4 Centre for Health Systems and Safety Research, Australian Institute of Health Innovation, Macquarie University, Sydney, NSW Australia; 5 Faculty of Pharmacy, University of Sydney, Sydney, NSW Australia; 6 Centre for Education and Research on Ageing, Concord Repatriation General Hospital, Sydney, NSW Australia

**Keywords:** Gout, Management, Pharmacist, Community pharmacists, Role, Medication adherence, Education

## Abstract

**Background:**

Gout is an increasingly prevalent form of inflammatory arthritis. Although effective treatments for gout exist, current management is suboptimal due to low medication adherence rates and treatments that are non-concordant with guidelines. Medications are the mainstay and most effective form of gout management. Thus, there is potential for community pharmacists to play an important primary health care role in gout management, however their current role and their potential to improve management of gout treatment is currently unclear. The purpose of the study is to explore the views of Australian pharmacists on their roles in gout management and to identify factors influencing their involvement in gout management.

**Methods:**

A convenience sample of community pharmacists were invited to participate using a snowballing recruitment strategy. Semi-structured, face-to-face interviews were conducted with 15 pharmacists of varying age, gender and pharmacy experience. Interviews focused on pharmacists’ experiences of managing gout, interactions with people living with gout and their perceived roles and responsibilities in gout management. Interviews were transcribed verbatim and independently analysed by two reviewers to identify themes.

**Results:**

The main role of pharmacists reported in gout management was providing patient education. The greatest facilitator to pharmacists involvement in gout management was identified to be pharmacists’ good understanding of gout and its management. Barriers to pharmacists involvement were identified to be difficulties in monitoring adherence to gout medications, low priority given to gout in the pharmacy compared to other chronic health conditions, and lack of specific training and/or continuing education in gout prevention and management.

**Conclusions:**

Pharmacists can expand their primary health care role in gout management, particularly in the area of ongoing provision of education to people living with gout and in monitoring medication adherence in patients. However, a number of barriers need to be overcome including difficulties in monitoring patient adherence to medications, ensuring a higher priority is given to chronic gout management and providing continuing training to community pharmacists about gout. Implications for pharmacist practice include initiating conversations about medication adherence and education when dispensing medications and undertaking continuing education in gout.

## Background

Gout is an extremely painful form of inflammatory arthritis characterised by high concentrations of uric acid (UA) in the blood and formation of monosodium-urate crystals within joints resulting in acute inflammation [1]. If left untreated, recurrent gout attacks can result in irreversible joint damage including cartilage loss and bone erosion [1]. Hyperuricaemia (serum UA concentrations > 0.42 mmol/L (7 mg/dL)) is the major risk factor for gout; the higher the UA, the greater the risk [2].

The incidence and prevalence of gout is increasing worldwide [3–5]. For example, recent reviews of Australian data suggested that the prevalence of gout has progressively increased with one study demonstrating it was 0.2% in 1968 [6], and an updated study reporting gout prevalence as 1.5% in 2013 [7]. In part, this can be attributed to the ageing population, as the prevalence of gout increases with age [7]. A number of international guidelines have outlined management strategies for gout, incorporating both pharmacological and non-pharmacological aspects [8–12]. Urate-lowering therapies (ULTs), e.g. allopurinol, are the main pharmacological treatments against gout and are typically required life-long to maintain lowered serum UA and reduce the risk of acute attacks [12]. Non-pharmacological interventions include lifestyle advice tailored to maintaining a healthy body weight and avoidance of excessive dietary purine content in food and beverages such as beer and selected seafood. Additionally, consumer education at the time of gout diagnosis is recommended [12].

Despite a good understanding of gout and the availability of effective treatments, current management of gout remains suboptimal [13]. In Australia, the majority of people living with gout are managed by their general practitioner (GP), who typically prescribe medications for gout management and provide some education about gout, diet and lifestyle [14]. However, despite GPs involvement in gout management, some people with gout do not receive optimal treatments concordant with guideline-based care [7, 14, 15]. Similarly, some people living with gout demonstrate suboptimal behaviour in their own gout management [16].

People living with gout have one of the lowest medication adherence rates for any chronic health condition, with one study reporting medication adherence rates of only 35% after 1 year [17, 18]. A contributing factor is thought to be the lack of patient understanding of gout and its optimal management [14, 19]. Unsurprisingly, providing comprehensive education to people living with gout has led to improved clinical outcomes. In an observational study, detailed education and close follow-up in people with gout by a research nurse contributed to beneficial outcomes (92% of participants achieved UA concentrations ≤0.36 mmol/L (6 mg/dL) in 12 months and 65% reported fewer acute gout attacks than the previous year) [20].

In Australia, community pharmacy is an integral component of the healthcare system by providing timely access to medicines and services that promote quality use of medicines. The role of community pharmacists involves dispensing of medications, provision of education about medications, identification of medication-related issues, medication reviews, and the provision of screening and preventive health care services for patients with chronic health conditions, such as diabetes screening and blood pressure monitoring [21, 22]. Australian pharmacists are currently unable to prescribe ‘prescription only’ medications, renew or change prescriptions (without authorization of a prescriber), order laboratory tests or easily access patients’ medical records. Increasingly, community pharmacists and GPs work more closely in managing people with chronic health conditions, although this has not always been the case [21, 23].

Community pharmacists could potentially improve primary health care management of gout, particularly by enhancing patient adherence to pharmacotherapy for gout. Studies have shown that pharmacists can improve medication adherence rates and therapeutic outcomes, as demonstrated in a number of chronic health conditions, such as diabetes [24, 25]. Studies from the United Kingdom demonstrated that two simple follow-up consultations with pharmacists providing support for patients with chronic illnesses for problems or concerns regarding their treatment, significantly improved medication adherence [24, 26]. In a United States study in people living with gout, 35% of individuals receiving an intervention from a clinical pharmacist achieved UA concentrations ≤0.36 mmol/L (6 mg/dL), compared to 13% of patients receiving usual care by a primary care physician after 26 weeks [27]. This specific intervention involved provision of written education material, on-going support and adjustment of gout medications by clinical pharmacists [27]. Although resource intensive, this demonstrates the potential benefits that pharmacists can have on gout management.

No research to date has specifically investigated the current role of pharmacists in relation to the management of gout. The aim of this study was to explore the current role of Australian community pharmacists in gout management and to identify any perceived facilitators or barriers to community pharmacists in managing, monitoring and treating people living with gout.

## Methods

### Recruitment

A convenience sampling strategy was used to recruit pharmacists for interviews. Community pharmacists known to the research team were contacted via a letter of invitation. A senior researcher also circulated letters of invitation within their pharmacist network. A snowballing recruitment strategy was used, where participants were asked to provide contact details of other community pharmacists who might be interested in participating [28]. No more than two pharmacists from the same practice were interviewed.

### Participants

Theme saturation was achieved following 15 interviews. Eleven female and four male pharmacists were recruited with an average of 11 years of experience (range 1−38 years). Participants practiced in 11 different pharmacy practices located across five metropolitan regions of Sydney, New South Wales, Australia. Pharmacists explained that they had frequent contact with people living with gout, and dispensed some form of gout medication at least 2–3 times a week.

### Data collection

Prior to each interview, the purpose of the study was explained to participants and written consent was obtained. Interviews were semi-structured using an interview guide (Table 1). Interview questions focused on the participant’s experiences of managing gout, interactions with people living with gout and the perceived role and responsibilities of a pharmacist in gout management. Questions were written with the input from a rheumatologist (RD), a pharmacist (AM) and a qualitative researcher (MB). One-on-one interviews were conducted in a convenient location for each participant, were 20–30 min in duration and participants were reimbursed for their time. All interviews were conducted by one researcher, a Medical Science Honours student (AC). Interviews were practiced beforehand to ensure competence in interview delivery and adequate participant understanding of the questions. Interviews were de-identified, audio-recorded and transcribed verbatim. Transcripts were not returned to participants for comment.

**Table 1:** Interview guide

Theme	Question
About the pharmacist	How long have you been practising pharmacy? How long have you worked in community pharmacy?
About the pharmacist’s interaction with people living with gout	Do you often dispense medications used in the treatment and prevention of gout? What are they? What proportion of the scripts would be for allopurinol? What dosages do you typically see on the prescriptions? What advice do you provide customers about their gout medication and management? What educational material do you provide customers with gout? If someone presents to the pharmacy with the symptoms of gout, what do you do? Do you feel you know enough about gout and how it should be managed? What over-the-counter products would you recommend for people with gout?
About the typical person with gout in your pharmacy	What is your gout customer typically like? Do they have any co-morbidities? Are there common complaints and/or challenges that your customers have or encounter with their gout? Does it affect their adherence to prescribed medication or diet? If you notice that a customer doesn’t return for a repeat allopurinol prescription regularly, what advice do you provide? Are there actions that you or your pharmacy takes to help people adhere to their medicines?
Referral to general practitioner	How often do you refer gout customers to see a doctor and/or get in contact with their GP yourself? What would make you do so?

### Analysis

Thematic analysis of transcripts was undertaken concurrently with conduct of interviews in order to determine theme saturation. Once theme saturation was achieved no further participants were invited to join the study [29]. Each transcript was analysed independently by two reviewers (AC, AN) using an inductive approach whereby researchers did not analyse transcripts with a pre-determined coding framework. Instead, after analysis of five transcripts, they convened to discuss emerging themes. Emerging themes were categorised into pharmacists’ current role and the barriers and facilitators to pharmacist involvement in gout management. A framework was then developed based on the themes that emerged. This framework was used for analysis of all subsequent transcripts and for reanalysis of the first five transcripts and allowed the most commonly appearing themes to be noted. Discrepancies between reviewers were resolved by consensus.

## Results

Pharmacist responses were grouped into three main areas, being the current role of pharmacists and the facilitators and barriers to their involvement in gout management, and summarised in Table 2.

**Table 2:** Summary of pharmacist roles, and facilitators and barriers to involvement, in gout management

Current roles	Facilitators	Barriers
• Provision of advice and education • Filling patients’ knowledge gaps • Medication adherence monitoring	• Good understanding of gout and its management	• Difficulty monitoring gout medication adherence • Lack of pharmacist training for gout • Gout viewed as less important than other conditions

### Current role of pharmacists in gout management

#### Advice and education

Pharmacists reported that they provided both pharmacological, diet and lifestyle advice to people with gout. However, only a small number of pharmacists reported that they provided education about the pathogenesis of gout. All pharmacists explained that they provided advice when they first dispensed gout medications but rarely thereafter. One pharmacist said, “*To be honest if they’ve used it before and they’ve got a history of using* [gout medications] *regularly I don’t generally give them advice”* (Pharmacist #95).

All pharmacists also reported providing some form of written educational material to people with gout, especially if it was the person’s first visit to the pharmacy or it was a new medication for the patient. Most commonly, this was the Consumers Medicines Information (CMI) for the medication being dispensed, which is a written plain language summary of Australian Therapeutics Goods Administration approved relevant information available for every prescription medicine in Australia. For example, “*First* [gout] *medication we offer CMI. But for repeat [scripts] we don’t offer CMIs anymore”* (#72).

#### Filling patients’ knowledge gaps

Most pharmacists felt that GPs often did not have time to provide sufficient education to people living with gout in a consultation. Consequently, pharmacists reported that the education they provided filled this gap in gout patients’ knowledge. For example, “*I think the majority of the time doctors don’t* [provide enough gout education] *and we’ll do it for them”* (#97).

#### Medication adherence monitoring

Most pharmacists interviewed acknowledged that monitoring patients’ adherence to gout medications was not something that was done actively or regularly, however it was recognised as something that should be done. One pharmacist said, “*Adherence* [to gout medications] *is one of those hard things. It’s not something that I look at on a general basis”* (#60), while another stated, “*it is meant to be a habit when we are dispensing scripts to see how often they’re getting their scripts filled… that’s a good indication to how compliant someone is”* (#95).

### Facilitators to pharmacists’ involvement in gout management

#### Good pharmacist understanding of gout and its management

The majority of pharmacists reported having a good understanding of gout medications and their appropriate use. For instance one pharmacist said about allopurinol use, “*Don’t start taking it if they are still having the gout attack, to wait until the gout settles and then they can start taking their allopurinol”* (#72). A few pharmacists also noted that allopurinol dose “*depends on renal function”* (#66). Pharmacists appeared to have sound understanding of the impact of diet and lifestyle on gout, noting “*Gout is really related to lifestyle measures and diet”* (#75) and gave appropriate advice regarding diet.

### Barriers to pharmacists’ involvement in gout management

#### Difficulty monitoring gout medication adherence

The most common barrier reported by pharmacists was the difficulty in monitoring patient adherence to gout medication. Tracking the intake of gout medications, particularly allopurinol, was said to be difficult from examination of the patient’s medication dispensing history because of the sizes of gout medication packs (60 units per pack). This made it hard for pharmacists to detect whether a gout patient was collecting their allopurinol scripts on time. One pharmacist noted, “*It’s not like a blood pressure tablet where you can see when they come in for their other monthly tablets that they’re missing out on their allopurinol. It takes a little bit more detection just to double check that it’s been 3 months”* (#66). A potential remedy for this raised by pharmacists was the use of electronic tools such as prompts within the dispensing history software to alert pharmacists. For example, one pharmacist stated, “*What makes it easier than ever is...we actually get a prompt if they’re not compliant”* (#80).

#### Lack of specific training in gout and its management

The majority of pharmacist participants reported a lack of specific professional training or continuing professional education in up to date gout management. For example, one pharmacist stated, “*There is no training* [in gout]*…I get a lot of information by drug reps for every disease under the sun except gout*” (#80). Another reported, “*The only training I’ve done on* [gout]*...was when the dosing recommendations changed”* (#95). Consequently, a small number of pharmacists stated that they “*pretty much rely on…university training”* (#66) or “*take it upon myself to do* [gout] *research”* (#80).

#### Gout viewed as being less important than other conditions

Most pharmacist participants reported that gout was a condition that was viewed with lesser importance than other chronic health conditions such as hypercholesterolaemia and cardiovascular disease. For example, a pharmacist said, “*We sort of look out more for statins...but not so much the allopurinol...don’t think of it as being as important”* (#91)*.* Subsequently, pharmacists felt that gout was not something that was given a lot of focus or high priority. One pharmacist stated, “*We may be thinking as we’re dispensing that he’s got more important conditions to be focusing on at the moment than his gout”* (#95).

## Discussion

This was the first qualitative study to explore the views of Australian community pharmacists regarding their role in the primary care management of gout. Pharmacists viewed their main role to be providing advice and education to people with gout. Participants demonstrated a good understanding of gout and how it is managed, which could facilitate a greater involvement in the management of people living with gout. The primary barrier to this was their reported difficulty in reliably monitoring adherence to gout medications.

Community pharmacies in Australia are one of the most accessible and convenient points of contact for patients within the healthcare system. This is because due to Australian Government regulations, pharmacies are widely geographically distributed and there is no fee or appointment required for consultations, which can be provided almost immediately [30]. Therefore, although GPs are the primary providers of care and management for people with gout, people visit their community pharmacist more often than their GP. Also contributing to this, is the need to refill repeat prescriptions for gout medications or more commonly, medications for comorbidities strongly associated with gout, such as hypertension [31]. The high accessibility, combined with frequent interactions, provides opportunities for pharmacists to be actively involved and expand their current role in gout management.

Studies have shown that some patients have a poor understanding of gout and its management [14]. This is thought to be due in part to inadequate consumer education provided at the point of diagnosis by their GPs [32]. Pharmacists in this study agreed with this, indicating that they were required to fill the patients’ gaps in knowledge about gout and associated medications. Interviews revealed that pharmacists have the knowledge and understanding to inform and educate patients about their gout medications, despite receiving limited training themselves. Patient understanding about medications used in gout management, especially the importance of good adherence, is crucial to optimal gout treatment. It has been suggested that some individuals feel more comfortable speaking with a pharmacist than a GP about their medications [30]. Therefore, an important opportunity exists for pharmacists to provide the much-needed education to people living with gout.

Despite pharmacists’ apparent good understanding of gout, participants reported that they do not always provide education to the patient. Interviews revealed that this was likely to be because of the low priority given to gout management and the limited training and continuing education reportedly received by pharmacists in this area. In Australia, registered community pharmacists have completed university level training and are required to maintain ongoing continuing professional education each year [33]. These findings implicate that it is important that pharmacists are provided with and engage in specific continuing education activities about gout. This would seemingly amplify the importance of gout, and increase the likelihood that pharmacists would provide ongoing patient education (rather than provision of education only at the beginning of treatment as reported in the interviews), which is needed for sustained health improvements [25, 34]. Furthermore, a recent review highlighted the need for more effective and comprehensive educational resources on gout and its management [35]. The development of such resources for use in patient-centred education in the pharmacy may also facilitate pharmacists to provide ongoing education and improve management. Such ongoing medication counselling is also a priority of the Pharmaceutical Society of Australia as outlined in their dispensing practice guidelines [36]. Therefore, it is suggested that pharmacists regularly initiate a conversation with the patient about their understanding of gout and the medication being dispensed at the time of dispensing gout medications.

In order for ULT to be optimally effective, it is imperative that people with gout adhere to medications in the long-term. But, adherence to gout medications is generally very poor [18]. Pharmacists are funded by the Australian Government to provide services such as medication adherence and medication management programs to patients [37]. Interviews revealed that pharmacists viewed monitoring patient adherence to medications as an important part of their role. However, they acknowledged that such monitoring is not routinely done for people taking gout medications. This is likely to contribute to the low medication adherence rates seen in gout. It is important for future practice that pharmacists take the time to check that patients are filling their gout scripts on time and initiate a conversation with the patient about their adherence to their gout medications. Pharmacists did, however, identify a range of factors that made monitoring of adherence to gout medications particularly difficult. This was thought by pharmacists to be primarily due to the large (government subsidised) pack sizes of allopurinol tablets (200 units per pack for 100 mg formulation, 60 units per pack for 300 mg formulation), resulting in lengthy periods between prescription refills. Participants suggested that the availability of electronic systems (e.g. alerts/reminders within the dispensing history software to notify the pharmacist when a patient’s script is overdue) would facilitate monitoring adherence. Similar electronic systems have been studied in community pharmacy and have been shown to be effective in preventing inappropriate prescribing and drug interactions and increasing the likelihood of pharmacists undertaking targeted education [38–40]. However, electronic systems to monitor medication adherence are not in widespread use. Thus, the development of ways to seamlessly integrate such electronic systems within the day-to-day workings of pharmacies could help pharmacists better monitor adherence. Furthermore, such a system also has the potential to fall within the Australian Government funding agreements with community pharmacies, further incentivising its use [37]. The regular use of electronic systems that provide pharmacists with alerts or reminders about overdue scripts would facilitate them to combat the low medication adherence rates seen in gout and lead to improved clinical outcomes.

### Limitations

In this study, there was reliance on self-reporting, as confirmation of the validity of pharmacist claims through additional studies was not performed. There is also an inherent selection bias when undertaking research that requires recruitment of participants. Individuals who agreed to the study may have been more interested in and/or knowledgeable about gout. However, undertaking continuous recruitment until theme saturation occurs can minimize selection bias. It would also be beneficial to investigate the views of people living with gout about the role of pharmacists, as well as the views of other healthcare providers involved in the management of gout, such as dietitians and GPs.

## Conclusions

This study explored the current role, and barriers and facilitators to involvement of Australian community pharmacists in gout management. Currently, pharmacists report playing a moderate role in assisting patients with managing this condition, mainly providing advice and education. Pharmacists’ good understanding of gout was seen to facilitate their involvement in gout management, but their lack of training and consideration for gout, together with difficulties in monitoring adherence to gout medications, prevented further involvement in its management. In this study, areas of opportunity to expand pharmacists’ role in gout management were also identified with the potential to lead to significant improvements in gout management in the community. These future implications for pharmacist practice include providing targeted ongoing education to people living with gout each time gout medications are dispensed, actively undertaking continuing education in gout, initiating conversations at the point of dispensing with the patient about their medication adherence, actively checking gout medication dispensing history and integrating electronic systems into pharmacies to assist with monitoring medication adherence.

## Abbreviations

CMI: Consumers medicines information
GP: General practitioner
UA: Uric acid
ULT: Urate-lowering therapy
